# Low frequency aeration of pig slurry affects slurry characteristics and emissions of greenhouse gases and ammonia

**DOI:** 10.1016/j.biosystemseng.2017.04.011

**Published:** 2017-07

**Authors:** Salvador Calvet, John Hunt, Tom H. Misselbrook

**Affiliations:** a Universitat Politècnica de València, Institute of Animal Science and Technology, Camino de Vera s.n., 46022 Valencia, Spain; b Rothamsted Research, North Wyke, Okehampton, Devon EX20 2SB, UK

**Keywords:** Gas emissions, Slurry mixing, Ammonia, Methane, Nitrous oxide, Carbon dioxide

## Abstract

Low frequency aeration of slurries may reduce ammonia (NH_3_) and methane (CH_4_) emissions without increasing nitrous oxide (N_2_O) emissions. The aim of this study was to quantify this potential reduction and to establish the underlying mechanisms. A batch experiment was designed with 6 tanks with 1 m^3^ of pig slurry each. After an initial phase of 7 days when none of the tanks were aerated, a second phase of 4 weeks subjected three of the tanks to aeration (2 min every 6 h, airflow 10 m^3^ h^−1^), whereas the other three tanks remained as a control. A final phase of 9 days was established with no aeration in any tank. Emissions of NH_3_, CH_4_, carbon dioxide (CO_2_) and N_2_O were measured. In the initial phase no differences in emissions were detected, but during the second phase aeration increased NH_3_ emissions by 20% with respect to the controls (8.48 vs. 7.07 g m^−3^ [slurry] d^−1^, P < 0.05). A higher pH was found in the aerated tanks at the end of this phase (7.7 vs. 7.0 in the aerated and control tanks, respectively, P < 0.05). CH_4_ emissions were 40% lower in the aerated tanks (2.04 vs. 3.39 g m^−3^ [slurry] d^−1^, P < 0.05). These differences in NH_3_ and CH_4_ emissions remained after the aeration phase had finished. No effect was detected for CO_2_, and no relevant N_2_O emissions were detected during the experiment. Our results demonstrate that low frequency aeration of stored pig slurry increases slurry pH and increases NH_3_ emissions.

## Highlights

• The effect of aerating pig slurry during 2 min every 6 h was tested.
• Aeration increased slurry pH by 0.7 units and NH_3_ emissions by 20%.
• CH_4_ emissions decreased by 40% and no significant N_2_O emission was detected.
• Mechanisms affecting slurry changes and emissions are discussed.

## 1 Introduction

In recent years, the growth in intensive pig production has lead the increasing global livestock production (FAOSTAT, 2016). However, intensive production of pigs tends to be decoupled from other agricultural production systems, and therefore a proper management of slurry becomes essential to avoid environmental impacts and to enhance nutrient recycling (Brockmann, Hanhoun, Négri, & Hélias, 2014). During their storage, pig slurries emit considerable amounts of ammonia (NH_3_) and greenhouse gases to the atmosphere, mainly in the form of methane (CH_4_). In intensive pig production, slurry treatment techniques become essential to fulfil the environmental regulations (e.g. Industrial Emissions Directive in Europe). These techniques are devised to facilitate slurry management, reduce the environmental impacts or obtain potential benefits such as high value fertilisers or biogas (Burton & Turner, 2003). However, potential side effects of certain treatments, such as nitrous oxide (N_2_O) emissions from aerobic treatments, must be accounted for.

Aeration has long been proposed as a treatment technique to reduce the nitrogen and organic matter loads of slurries, and thus reduce the pollution risk (Bicudo and Svoboda, 1995, Bicudo, 1995, Burton, 1992, Cumby, 1987). During the aeration of animal slurries, the anoxic and reductive conditions in which slurries are normally stored (e.g. <1 g L^−1^ dissolved O_2_) change to aerobic and oxidising conditions (e.g. >4 g L^−1^ dissolved O_2_) (Cheng & Liu, 2001). Also, aeration normally involves stirring the slurries, both avoiding sedimentation and crust formation. This changes the biochemical and microbial reactions in the slurry, depending on the aeration set-up parameters (for example aeration flow rates, frequency and duration in case of intermittent aeration, tank size and air bubble size, among others).

The effect of aeration on the fate of nitrogen has been long demonstrated (Béline and Martinez, 2002, Béline et al., 1999, Cheng and Liu, 2001). These studies have revealed that using appropriate aeration flows in the case of continuous reactors or intermittent aeration for batch reactors achieves high nitrogen removals as a consequence of nitrification and denitrification processes, producing nitrogen gases. Therefore, research has recently focused on reducing N_2_O emissions by controlling the process variables including intermittent aeration or aeration flow rates (Castro-Barros et al., 2015, Hu et al., 2010, Hu et al., 2011a, Hu et al., 2011b, Hu et al., 2011a, Hu et al., 2011b, Lackner et al., 2014, Mampaey et al., 2016, Pan et al., 2014, Wang et al., 2016). According to these authors, changing operation parameters leads to N_2_O emission factors mostly between 1% and 10% of the initial N.

Aerobic treatment systems are not considered best available techniques because they involve a loss of a valuable nutrient and the side effects on N_2_O emissions are still considerable (European Commission, 2015). However, the short time aeration (less than 1% of time) of slurries could have the potential to reduce ammonia emissions without these side effects, although to the authors' knowledge, very limited research has been conducted in this area. In this process, two effects are expected to occur: slurry mixing and partial oxygenation. The mixing effect is the main hypothesis for reduced NH_3_ emissions. As first suggested by Ni, Hendriks, Vinckier, and Coenegrachts (2000) and reviewed by Hafner, Montes, and Alan Rotz (2013), CO_2_ emission from slurry increases surface pH, thus enhancing NH_3_ emissions. As a consequence, NH_3_ emissions during and immediately after aeration may be lower than untreated slurries. However, it has also been reported that the aerobic treatment of manure increases slurry pH on average, as a consequence of the removal of volatile fatty acids (Fangueiro et al., 2015, Sørensen and Eriksen, 2009, Zhang and Zhu, 2005). Therefore, potential effects of short aeration times on pH and NH_3_ emissions must be evaluated by research.

The partial oxygenation caused by low frequency aeration may have potential effects on CH_4_ and N_2_O emissions. Research on aerobic treatment of slurries normally considers a longer duration of the aerobic phase in comparison to the anaerobic phase, and results indicate that nitrification and denitrification is enhanced, whereas methanogenesis is inhibited (Castro-Barros et al., 2015, Cheng and Liu, 2001, Hu et al., 2010, Hu et al., 2011a, Hu et al., 2011b, Hu et al., 2011a, Hu et al., 2011b, Lackner et al., 2014, Wang et al., 2016). However, no information is available on how these emissions are affected by low aeration frequency.

The objectives of this study are: first, to determine whether a low frequency aeration (2 min each 6 h) of pig slurry affects its composition and the related emissions of NH_3_, CH_4_, N_2_O and CO_2_; and second, to analyse and describe the mechanisms underlying these changes.

## 2 Methodology

### 2.1 Experimental design

The experiment was conducted at the Rothamsted Research North Wyke site from 8th June to 22nd July 2016. A slurry storage system similar to that described by Misselbrook, Hunt, Perazzolo, and Provolo (2016) was used, comprising six 1.25 m^3^ tanks (1.20 m diameter and 1.12 m height) located in a covered area to exclude rainfall. Slurry was obtained from a local commercial finishing pig farm. Slurry stored for about 8 weeks was collected from the below slat storage and transported to the experimental site using a 6 m^3^ slurry tank. Slurry was mixed and then representatively divided among the experimental tanks, and thus each tank was filled with 1 m^3^ of slurry (88 cm depth). Three of the tanks were subjected to the aeration treatment, whereas the other three tanks were not treated and served as control tanks. Slurry composition and gaseous emissions were monitored during the experiment.

**Fig. 1:**
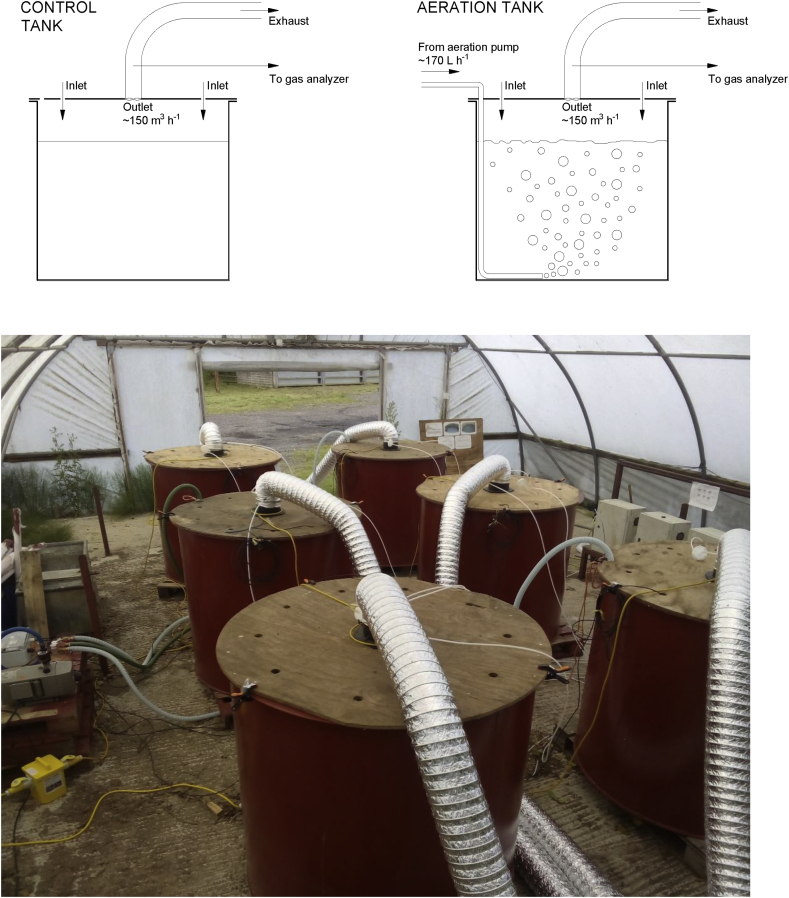
Slurry tanks used in the experiment. Schematic of the control and aerated tanks (upper) and arrangement in the polytunnel (lower).

The experiment consisted of three phases. The first phase lasted for the first 7 days of storage and all tanks were subjected to the same management, that is, none of the tanks was aerated. During this phase, potential differences among tanks were evaluated. The second phase lasted for 4 weeks during which aeration was conducted in the aeration treatment tanks. The aeration system consisted of a low frequency injection of air. Each slurry tank under the aeration treatment had 170 L min^−1^ of ambient air injected over 2 min every 6 h. Injection was programmed to occur at 03:00 h, 09:00 h, 15:00 h and 21:00 h. An automatic controller was designed specifically to control operation times, and air was injected using a pump (Becker VT 4.10, Wuppertal, Germany). Air was injected at the bottom of each tank through a 32 mm diameter PVC pipe. Details of the aerated and non-aerated tanks can be seen in Fig. 1. Finally, in a third phase over the last 9 days of the experiment, no aeration was conducted in any of the tanks, aiming to evaluate potential permanent changes in slurry composition and gaseous emissions after the aeration treatment had finished. From each tank, representative slurry samples were taken for analysis at the beginning of each phase.

### 2.2 Slurry characteristics

Slurry samples were taken at the start of each storage phase and analysed for total solids and volatile solids content, total nitrogen (N), ammonium-N, and pH, following a similar procedure to Misselbrook et al. (2016). Total solids content was determined by measuring the mass loss after drying at 85 °C for 24 h. Volatile solids content was determined on a subsample of the total solids by measuring the mass loss on ignition at 550 °C. To determine total N content, samples were pipetted and weighed into tin capsules containing liquid absorbing pads. These were then analysed on an elemental analyser (Carlo Erba, NA2000, Milan, Italy) connected to a 20–22 isotope ratio mass spectrometer (Sercon, Crewe, UK). Ammonium-N was determined by automated colorimetric analysis following extraction with 2 M KCl. Total oxidised nitrogen analysis was carried out with an extraction using 2 M KCl and determined by the colorimetric vanadium chloride method.

As pH was hypothesised to be a key driver for NH_3_ emissions, it was monitored 3 times per week using a portable meter with pH probe (HI 9025, Hanna Instruments, Leighton Buzzard, UK). Measurements were conducted throughout the experimental period at the slurry surface and at a depth of 10 cm. During phases 1 and 3, measurements were conducted once per measurement day, whereas during the aeration phase (phase 2) the measurements were repeated 3 times: one 10 min before aeration, another immediately after aeration, and the final 15 min after aeration. Ambient and slurry temperature of each tank (at a depth of 40 cm) was measured every minute using an automated data logger (Grant Data Acquisition Series 2040, UK). Changes in slurry temperature due to aeration were therefore monitored.

In addition, the CH_4_ producing potential (B_o_) of the slurry at the start of each storage phase was determined using an automated laboratory incubation system (AMPTSII, Bioprocess Control, Lund, Sweden). Slurry samples were incubated at 37 °C with an inoculum obtained from a farm-fed mesophilic anaerobic digester. To reduce direct production of biogas from the inoculum, it was incubated in advance for 5 days at 37 °C to deplete residual biodegradable organic material present. Samples were prepared for incubation in 500 mL reactor bottles; each bottle contained 400 g of inoculum and slurry substrate at a 2:1 ratio by mass of inoculum VS to substrate VS. Gas generated from the incubation reactor bottles passed through a 3 M NaOH solution (with thymolphthalein pH indicator) to remove CO_2_ and H_2_S gas, leaving only CH_4_ to pass through the gas volume measuring device, which operates on a principal of buoyancy and liquid displacement. Blank samples consisting of just inoculum were included. The reactor bottles were stirred automatically at 110 rpm for a period of 60 s every 2 min. The system was controlled via a PC, and the gas flow rate and cumulative gas volume produced from each reactor were normalised for temperature and pressure and recorded continuously for 45 days.

### 2.3 Gaseous emission measurements

The slurry storage tanks were fitted with specially adapted lids, which had a central circular hole of 10 cm diameter to which a fan was fitted to draw air from the tank headspace (Fig. 1). Air was drawn into the tank headspace via ten holes around the outer edge of the lid each of 3 cm diameter. The air was exhausted from the tank headspace by the fan through a duct to an area outside the shed. The lids were left in-situ throughout the storage period with fans running continuously. Air flow rate was nominally 150 m^3^ h^−1^, but was measured at the duct outlet for each tank three times per week. The tanks with lids therefore acted as large dynamic chambers for emission measurements. Gas concentration measurements were made via a cross-sectional sampling tube within the outlet duct of each tank. Inlet concentrations were also measured and at two places within the shed. Estimates of flux for each gas (*F*, g h^−1^) was made according to the following equation:

$$F=\frac{V\left(C_{o}-C_{i}\right)}{1000}$$

where V (m^3^ h^−1^) is the air volume flow rate and C_o_ and C_i_ the outlet and inlet gas concentrations (mg m^−3^), respectively.

Gas concentrations were measured using Los Gatos analysers (Model 911-0016 for NH_3_ and 915-0011 for CH_4_ and CO_2_, Los Gatos Research, California) based on cavity enhanced absorption spectroscopy, with a multiport inlet unit (Model 908-0003-9002, Los Gatos Research, California). Sampling was on a semi-continuous basis with measurements from each sampling position (6 tank duct outlets and 2 ambient air sampling positions). Two sampling protocols were established for the different phases of the experiment. During the first and third phases, no aeration was conducted and each sampling position was monitored for 7.5 min. The analyser continuously cycled around the eight sampling positions, thus completing a measurement cycle in 1 h. The instrument measured gas concentrations every 10 s and equilibration of the concentration reading when switching between sampling points was about 3 min. The mean concentration at each sampling point for a given cycle was derived from the last 3.5 min of readings (20 concentration measurements) at each sampling point, discarding the initial 4 min of readings. During the second phase when aeration took place every 6 h, the cycle described before was programmed to take place every 2 h, alternating with 1 h of continuous monitoring of an aerated tank (from 10 min before aeration began to 48 min after aeration finished). Each tank was monitored in this way during two days once a week. This was devised to determine the emission profile during and after aeration.

Ammonia concentration measurements made by the Los Gatos analyser were checked against those made by sampling air through acid absorption flasks twice per week throughout the experiment. These latter measurements were made over a 6-h period by subsampling the air flow from the tank outlet ducts or from the ambient sampling points and passing through acid absorption flasks (100 mL of 0.01 M orthophosphoric acid per flask). The quantity of ammonia-N trapped in the absorption flasks was determined by automated colorimetry and was divided by the volume of air passing through the flask to derive the concentration in the sampled air. A linear correspondence was found between the automated measurements and acid trapping of NH_3_, and thus all automated measurements taken with the Los Gatos analyser were corrected accordingly.

Nitrous oxide concentrations were obtained by taking gas samples manually from the tank outlet ducts and ambient sampling points, storing in evacuated glass vials and analysing by gas chromatography (GC; Clarus 500, Perkin Elmer, Buckinghamshire, UK). Samples were taken on two occasions per week. The same samples were also analysed for CH_4_ and CO_2_ concentration by GC, which provided data for verification of the continuous analyser. During the first and third phase, when no aeration was conducted, only one gas sample was collected per tank. During the second phase of the experiment, three samples were collected per tank (one 10 min before, one during, and one 15 min after aeration).

Hydrogen sulphide (H_2_S) was measured for two days using detection tubes (Draeger Safety). Spot measurements were conducted two times during the aeration phase, both in the aerated and non-aerated tanks, to obtain an approximate value of the potential emission of this gas due to the aeration and mixing process.

### 2.4 Data treatment and statistical analysis

The dataset of continuous emission fluxes was integrated to obtain daily emissions (g day^−1^) of each gas. Daily averages of control and treated tanks were obtained and are presented to describe the temporal evolution of emissions. A two-way analysis of variance was conducted considering phase, treatment and their interaction as factors explaining differences in slurry composition and emissions. Differences were considered statistically significant at P < 0.05. The statistical analysis was conducted using SAS.

During the second phase, the immediate effect of aeration on slurry temperature and emissions was analysed in qualitative terms; measured values were integrated into a 1-min time resolution and compared to values immediately before aeration.

## 3 Results

### 3.1 Evolution of slurry characteristics

The slurry composition and characteristics at the start of each phase are presented in Table 1. Slurry composition (dry matter, volatile solids, nitrogen content, ammonia content and methane potential) did not differ among tanks assigned to different treatments at the start of the experiment, and also at the start of phase 2, before aeration was initiated. After phase 2, dry matter content of slurry increased in the control tanks with respect to the aerated tanks, whereas no differences were found for the rest of slurry properties. Nitrate and nitrite content of the slurry was negligible compared to total N content (lower than 1 mg L^−1^) throughout the experiment.

**Table 1:** Slurry characteristics at the start of each phase (total solids, volatile solids, total N, ammonium N, nitrate and nitrite). Methane potential (B_0_) is also provided for the initial slurry. Average values of pH, temperature and gas emissions during each phase are also provided. Within each phase, values with different letters in the same row indicate statistical differences between treatments (P < 0.05). Standard errors (S.E.) and P-values for the effects of Treatment (T), phase (P) and their interaction (T × P) are also indicated. Non-significant differences (n.s.) are considered for P > 0.05.

	Phase 1		Phase 2		Phase 3		S.E.	P-values		
	Control	Aeration	Control	Aeration	Control	Aeration		T	P	T × P
Total solids (TS, g kg^−1^)	2.49^a^	2.60^a^	2.85^ab^	2.72^a^	3.14^b^	2.74^a^	0.12	n.s.	0.023	n.s.
Volatile solids (g kg^−1^ TS)	675	683	690	678	690	672	8.9	n.s.	n.s.	n.s.
Total N (g kg^−1^)	3.97	4.12	4.10	4.26	4.13	4.04	0.09	n.s.	n.s.	n.s.
Ammonium N (g kg^−1^)	3.02	3.03	2.93	2.97	3.07	2.93	0.04	n.s.	n.s.	n.s.
B_o_ (m^3^ CH_4_ kg^−1^ VS)	0.76	0.71	0.64	0.70	0.60	0.69	0.04	n.s.	n.s.	n.s.
pH at 10 cm depth	7.06^a^	7.03^a^	7.05^a^	7.50^b^	7.09^a^	7.65^c^	0.02	<0.001	<0.001	<0.001
Slurry temperature (°C)	16.10^abc^	16.18^ab^	15.68^a^	16.03^ab^	17.68^bc^	17.88^c^	0.61	n.s.	0.015	n.s.
NH_3_ emission (g m^−3^ day^−1^)	7.14^a^	6.81^a^	7.07^a^	8.48^b^	9.35^b^	11.19^c^	0.38	0.009	<0.001	0.034
CH_4_ emission (g m^−3^ day^−1^)	4.33^a^	4.24^ab^	3.39^b^	2.04^a^	4.99^a^	1.62^a^	0.28	<0.001	<0.001	<0.001
CO_2_ emission (g m^−3^ day^−1^)	120.5^d^	107.1^cd^	90.4^b^	97.3^bc^	85.6^b^	67.9^a^	4,8	n.s.	<0.001	n.s.

**Fig. 2:**
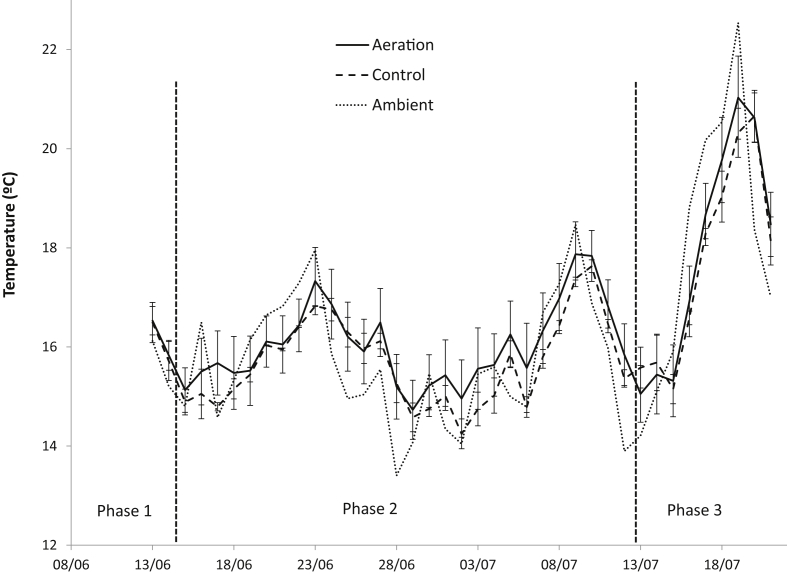
Evolution of daily temperature (left) for aeration tanks (), control tanks () and ambient temperature (). Evolution of pH (right) at 10 cm depth of aeration () and control tanks (). pH measured at 1 cm depth is also indicated for aeration () and control tanks (). Standard errors of means are provided as error bars (n = 3). The phases of the experiment are also indicated: Phase 1: no aeration conducted in any tank; Phase 2: aeration conducted only in the aeration tanks; Phase 3: no aeration conducted in any tank.

Daily average ambient temperatures ranged from 13.4 °C on 28th June to 22.5 °C on 19th July, and ranged mostly from 14 to 18 °C (Fig. 2). As the shed had no climate control, tank temperature oscillated with ambient temperature, and therefore changed between days and followed a sinusoidal diurnal pattern with highest temperatures in the afternoon and lowest temperatures early in the morning. Within each phase, average slurry temperatures did not differ significantly between treatments, as shown in Table 1.

**Fig. 3:**
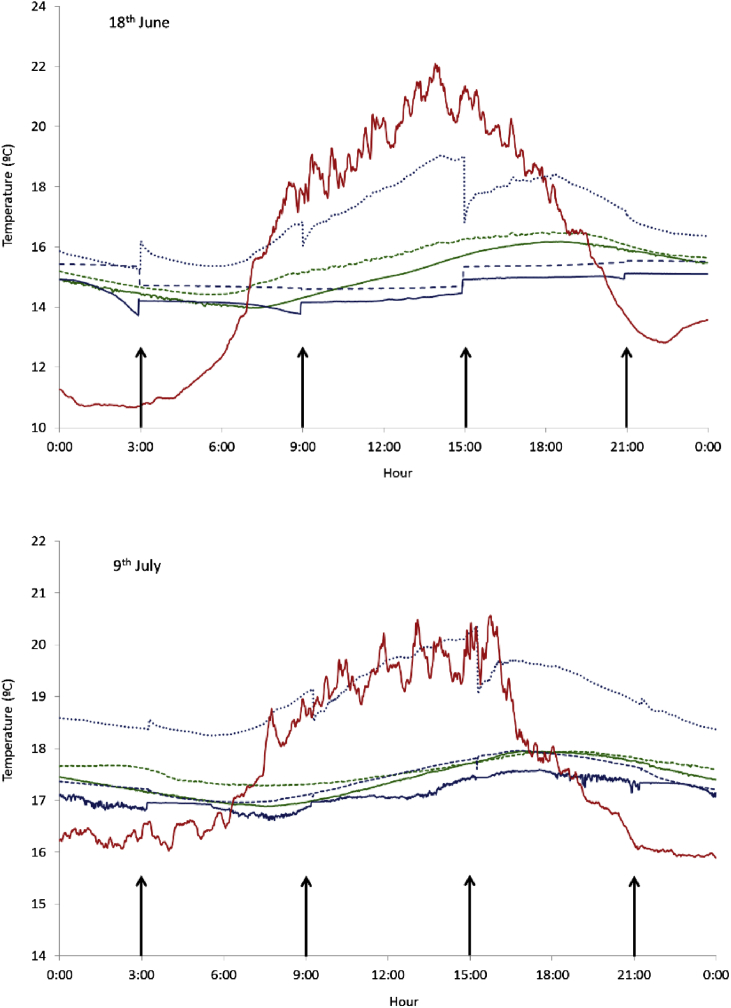
Daily variation in slurry temperature across 2 day at the beginning (18th June) and at the end (9th July) of the aeration phase. Control tanks are shown in green (Control 1 in solid line, Control 3 in dashed line). Aeration tanks are shown in blue (Aeration 1 in solid line, Aeration 2 in dashed line, Aeration 3 in dotted line). Ambient temperature is also shown in red. The four aeration events during the day are indicated with arrows.

The aeration had an immediate but non-permanent effect on slurry temperature. Changes from an increase of 0.6 °C to a decrease of more than 1 °C were detected immediately after aeration compared to before aeration. However, this effect was slight and inconsistent, and therefore no clear pattern was obtained. The changes in slurry temperature varied among tanks, and depended on the time of the day and on the day of experiment. Temperature changes tended to be higher during the first aeration days than during the last ones. Figure 3 shows the daily evolution of temperature and temperature changes in the aerated tanks at the aeration moments.

No statistical differences in pH were found between treatments during the initial phase where no aeration was conducted in any tank. However, during phase 2 pH was significantly higher for the aerated than for the control tanks (Table 1). The average initial pH of all tanks during this phase was 7.05. However, as shown in Fig. 2, pH increased steadily during the first two weeks of aeration until it reached values around 7.7. In contrast, the pH of control tanks remained stable at around 7.1. In phase 3, when aeration had finished, the pH of the aerated tanks tended to decrease, but after 10 days of no aeration it was still about 0.5 pH units higher than in the control tanks, which remained constant.

Surface pH (1 cm depth) is also shown in Fig. 2. Some difficulties were found when measuring surface pH, since a crust formed during phases 2 and 3 in the control tanks. For this reason, surface pH values were very variable and thus unreliable. Despite having a higher variability, surface pH was always higher at 1 cm depth than when measured at 10 cm depth. Differences in pH between 1 and 10 cm depth were about 0.3 units in all treatments for the first phase, when no aeration was conducted on any tank. During phase 2 for the aeration treatment, the difference between surface and 10 cm depth surface decreased with time as pH at 10 cm increased, and at the end of the experiment this difference was about 0.05 units. In the control tanks this difference also lowered during phase 2, and was generally between 0.1 and 0.2 units.

The loss of slurry volume at the end of the experiment, compared to the start, was similar among all treatments and was about 10% of the initial volume.

### 3.2 Gas emissions

For NH_3_ concentrations, the comparison between the automated analyser and the reference acid trapping method showed a linear relationship (R = 0.96, P < 0.05, details not shown) within the range of concentrations measured (between 0 and 6 mg m^−3^). However, the automated system overestimated the results from the acid trapping by 54%, and this bias was therefore corrected before data analysis. For CH_4_ and CO_2_ measurements, no significant differences were detected between the automated measurement system and the analysis of sample gases by gas chromatograph.

No statistical differences between treatments were found for NH_3_, CO_2_ or CH_4_ gaseous emissions during the first phase, when no aeration was conducted at any tank (Table 1). However, aeration during phase 2 significantly increased NH_3_ emissions by 20% and reduced CH_4_ emissions by 40%. This effect was not only detected during phase 2, but also during phase 3, after aeration had finished.

**Fig. 4:**
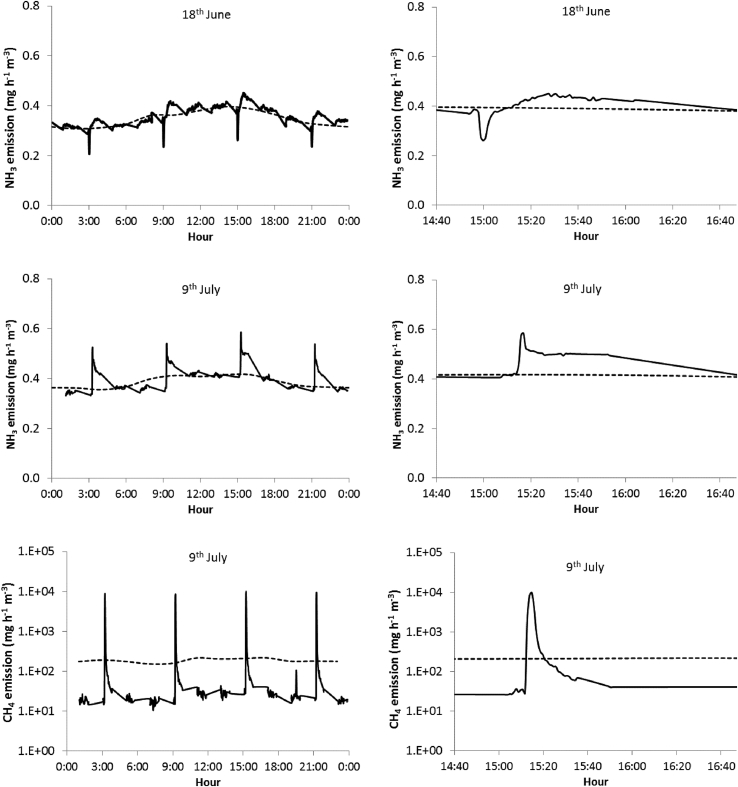
Example of the effect of short term aeration on NH_3_ emission at the start of phase 2 (18th June, upper) and at the end of phase 2 (9th July, centre). On the left, the daily variation in emissions in one of the aerated tanks (solid line) and the average emission for all control tanks (dashed line) are plotted. On the right, the detail across one aeration event is provided. The effect of aeration on CH_4_ emissions is also plotted for 1 day at the end of phase 2 (9th July, lower, please note the logarithmic scale).

**Fig. 5:**
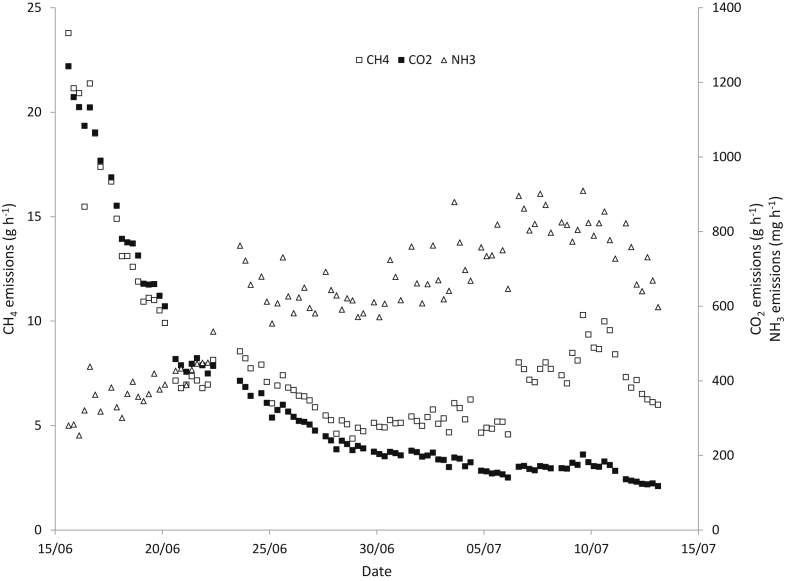
Evolution of emission fluxes of NH_3_ (triangles) CH_4_ (crosses) and CO_2_ (rectangles) during the aeration events, during phase 2.

**Fig. 6:**
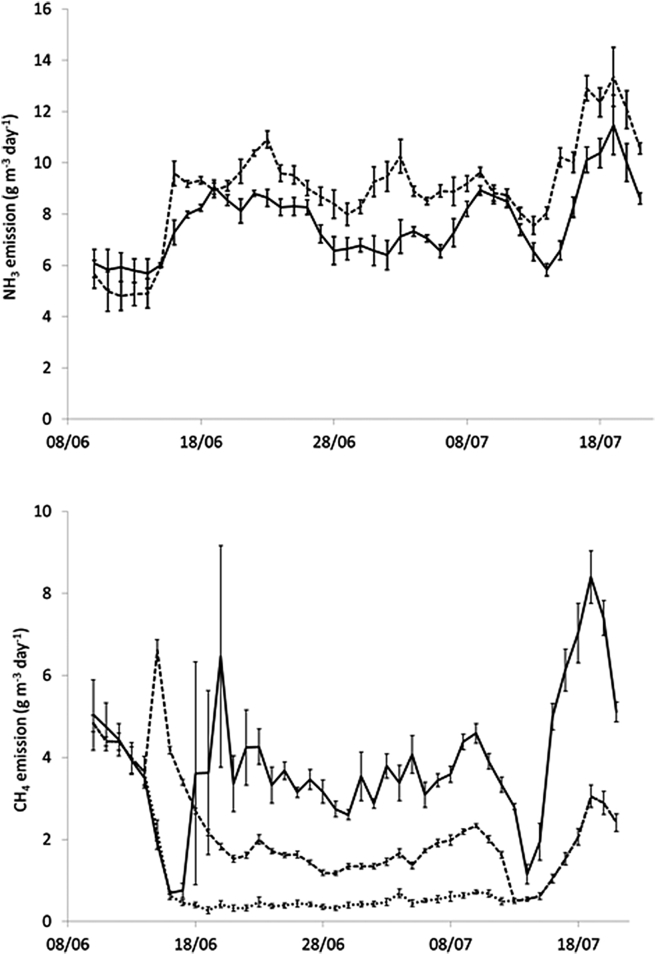
Daily average emissions of NH_3_ (upper) and CH_4_ (lower) during the experiment. Emissions from control tanks are represented with solid lines and from aeration tanks with dashed lines. For CH_4_ emissions from aeration tanks, during phase 2 two values are provided. Firstly, the emission accumulated during the whole day except for 16 min during and after aeration periods (dotted line); and secondly, total emissions when adding the release during and after aeration (dashed line).

The short term effects of aeration on NH_3_ and CH_4_ emissions are shown in Fig. 4, where the daily evolution of emissions is presented. Ammonia emissions followed a daily sinusoidal pattern in accordance with temperature variation. During the first days of phase 2, NH_3_ emission did decrease during and immediately after aeration, but after a few minutes it recovered to initial values, or even higher (Fig. 4). By the end of phase 2 emissions increased during the aeration and this increase remained after aeration ceased. These variations, however, accounted for a small share of total emissions because of their low magnitude and duration. Emissions during the 2-min aeration events increased slightly with time, as shown in Fig. 5, and at the end of phase 2 were approximately double with respect to the beginning of that phase. Overall, the average NH_3_ emissions during phases 2 and 3 were significantly higher in the aerated than in the control tanks (20% in both phases, Table 1 and Fig. 6).

Methane emissions tended to be stable throughout the day, and during phases 2 and 3 were higher in the control than in the aerated tanks (Fig. 6). However, aeration caused a sharp release of CH_4_ during the aeration event (Fig. 4). The emission during this short period was about 1000 times higher than the baseline emission. Despite the fact that these outbursts of CH_4_ lasted for short time, due to the high magnitude of the release in the aeration events, they contributed a major proportion of total CH_4_ emissions from the aerated tanks during phase 2. Between 70 and 80% of total CH_4_ emissions emitted from this treatment were produced during or in the few minutes following the aeration (Fig. 6). In the aerated tanks, both CH_4_ emissions during aeration and baseline emissions decreased exponentially over the first days of the second phase (Fig. 5), whereas the emission level of the control tanks was relatively constant (Fig. 6). Total CH_4_ emissions from aerated tanks were significantly lower than in the control tanks, not only in phase 2, but also in phase 3 (Table 1).

The behaviour of CO_2_ emissions followed a similar pattern to CH_4_ emissions, with very high emissions during and immediately after the aeration. Some problems with the analyser related to overnight measurements prevented the calculation of daily averages for all days, as obtained for NH_3_ and CH_4_, and therefore details are not shown. However, the evolution of emissions during the aeration events was monitored (Fig. 5), showing a very similar tendency to CH_4_. From the available data we found no significant differences, either for phase 2 or phase 3 (Table 1), while in general CO_2_ emissions decreased with time, being significantly lower in phase 3 compared with phase 1.

Nitrous oxide concentrations measured at the tank exhaust air were not significantly different from the inlet air at any tank and at any moment of the experiment, and therefore N_2_O emissions were considered to be negligible and not affected by the slurry treatment system. From the spot measurements of H_2_S, it was found that concentrations were lower than the detection limit of the colorimetric tubes used (<0.5 ppm) for the control tanks, and also for the aerated tanks when no aeration was being conducted. In contrast, during aeration a sharp increase in H_2_S concentration was found (between 60 and 80 ppm), which involved an emission of roughly 4–5 mg m^−3^ s^−1^.

## 4 Discussion

The aeration of stored slurry produced slight changes in the main chemical properties, significant changes in slurry pH, increased NH_3_ emissions, whereas increased oxygen supply reduced CH_4_ emissions. Furthermore, these effects showed an evolution over the weeks of the experiment. Predominantly, the low frequency aeration affected three main parameters (pH, temperature and crusting), resulting in very different behaviour of emissions with respect to the control tanks.

Aeration of slurry had a combined effect of mixing and oxygenating. As reported through previous research (Blanes-Vidal et al., 2012, Ni et al., 2000, Petersen et al., 2014), aeration initiated a mixing of the slurry and disturbed the pH gradient, thus reducing the pH at the slurry surface and thus the NH_3_ emissions. This effect was clearly detected only during the first days of treatment and had a very short duration, since emissions returned to similar levels after a few minutes. Despite the short duration, aeration increased the bulk pH over the medium term, presumably because of the degradation of volatile fatty acids, as reported previously (Fangueiro et al., 2015, Sørensen and Eriksen, 2009, Zhang and Zhu, 2005). According to our results, the medium term and permanent rise in pH had more influence on NH_3_ emissions than the short term and transitory decrease in surface pH. Furthermore, during the last days of the aeration phase very little difference was detected between surface and bulk pH, and as both were relatively high (about 7.7), NH_3_ was stripped to the atmosphere during the aeration events.

Crusting can also affect ammonia emissions. The aerated tanks were mixed during the aeration process four times a day, whereas control tanks were not stirred at all. Consequently, a crust formed in the control tanks (visual observation), which was not the case for the aerated tanks. Although the crust was removed during the slurry sampling in all tanks (at the beginning of each experimental phase), it re-established within a few days if no aeration was conducted. Since crust formation has been identified as a potential NH_3_ mitigation strategy (Misselbrook et al., 2005, Wood et al., 2012), the effect of crust removal could also have contributed to higher emissions in the aerated tanks.

Longer aeration times have been reported to increase slurry temperature as a consequence of the aerobic degradation of organic matter (Burton, 1992, Heinonen-Tanski et al., 1998). However, no reports are available on the effect of very short aeration times such as those used in this study. The temperature changes detected due to the aeration lead to no clear interpretation, because both increases and decreases of temperature were detected. The effect of aeration on temperature tended to be higher during the first days of aeration. This may indicate the effect of aerobic degradation processes, as more easily degradable organic matter may be expected to be present in the slurry at the beginning of the aeration phase. In contrast, temperature decreases were also detected, and this could be a consequence of enhanced water evaporation and sensible heat transfer during the aeration process. However, the energy removal by water vapour evaporation during the short time of aeration (only 2 min) was roughly estimated from measured values of temperature and relative humidity. These calculations (data not shown) indicate that the heat removed from water due to evaporation would decrease the slurry temperature by no more than 0.05 °C. The decrease in slurry temperature was double-checked with different temperature probes, so instrument malfunction was not considered to be a reason, and there may have been other influencing factors not considered in this analysis. In any case, temperature changes were of low magnitude (typically less than 0.5 °C), and therefore this effect was expected to be low compared to the more evident effects of pH changes in the medium term.

As expected, low frequency aeration of slurry decreased CH_4_. However, high amounts of CH_4_ were released during aeration events, leading to even higher emissions during the first days of treatment. As reported in the literature (Amon et al., 2006, Blanes-Vidal et al., 2012, Dai and Blanes-Vidal, 2013), the solubility of CH_4_ in water is very low, and therefore the CH_4_ produced in the slurry is kept in small bubbles before escaping to the atmosphere. During the first days of aeration, CH_4_ is still produced because easily degradable organic matter is still available, and low frequency aeration probably did not completely preclude the necessary anaerobic conditions. However, following low frequency aeration for several days, readily available organic matter such as volatile fatty acids tend to be aerobically degraded (Burton, 1992, Zhang and Zhu, 2006), thus reducing the potential for CH_4_ generation. On the contrary, aeration in this case was not related with increased N_2_O emissions. It seems that oxygen supply was not enough to enhance nitrification processes, which are required to emit relevant amounts of this gas. This is evidenced by the low content in oxidised species (nitrites and nitrates) in both control and aerated treatments.

It must be considered that this was pilot study conducted under specific conditions. Extrapolation of these results to real farm conditions must take into account differences in slurry volume (and surface area to volume ratio), slurry management strategies and ambient conditions. Changes in the scale, from pilot to farm slurry tanks, have been described for aerobic treatments (Sneath, Burton, & Williams, 1992) and these would be of relevance to our study because of differences in the volume and homogeneity of aeration and their derived effects (oxygenation and mixing). Although similar effects on slurry pH and emissions may be expected from tanks managed in a similar way as in this study, this could be confirmed with on-farm slurry tank measurements. On the contrary, differences may be expected for different slurry management strategies. This study mimics an external slurry store without addition of fresh slurry. This study is almost certainly not representative of under-slat slurry storage, where fresh urine and faeces are continuously added from animal excretions.

The behaviour of external slurry storages with frequent addition of fresh slurry may also be different because of the continuous incorporation of untreated slurry, for example in continuous reactors (Loyon, Guiziou, Beline, & Peu, 2007). Also, longer slurry storage times than in this study occur under farm conditions. Our results suggest that pH differences remain as long as the aeration phase continues, but pH may be reduced gradually after aeration finishes. Specific research would confirm the effects of aeration at longer times, but a very relevant decrease in NH_3_ emissions would be needed to counteract the initial increased emissions. Finally, changes in the ambient conditions (particularly the temperature) would probably affect these results. According to the literature, temperature affects directly the emissions of CH_4_ (Haeussermann, Hartung, Gallmann, & Jungbluth, 2006) and NH_3_ (Misselbrook et al., 2016). Therefore, it may be expected that the effects of low frequency aeration would increase with temperature. However, this must be confirmed with specific research.

In conclusion, short frequency aeration of stored slurry as tested in this study cannot be considered an option to mitigate NH_3_ emissions. Slurry mixing reduced the pH gradient in the slurry surface but this effect did not compensate the pH increase in the slurry, and therefore NH_3_ emissions increased. CH_4_ emissions decreased by approximately 40% in spite of the short duration of aeration. No N_2_O emissions were detected and therefore, this treatment reduced greenhouse gas emissions. The applicability of these results at different conditions to those in this study must be evaluated in practice.
